# Complex dystonias: an update on diagnosis and care

**DOI:** 10.1007/s00702-020-02275-y

**Published:** 2020-11-13

**Authors:** Rebecca Herzog, Anne Weissbach, Tobias Bäumer, Alexander Münchau

**Affiliations:** Institute of Systems Motor Science, University of Lübeck, Center for Brain, Behavior and Metabolism, Ratzeburger Allee 160, 23538 Lübeck, Germany

**Keywords:** Complex dystonia, NBIA, Infantile cerebral palsy, Diagnostic, Management, Red flags

## Abstract

Complex dystonias are defined as dystonias that are accompanied by neurologic or systemic manifestations beyond movement disorders. Many syndromes or diseases can present with complex dystonia, either as the cardinal sign or as part of a multi-systemic manifestation. Complex dystonia often gradually develops in the disease course, but can also be present from the outset. If available, the diagnostic workup, disease-specific treatment, and management of patients with complex dystonias require a multi-disciplinary approach. This article summarizes current knowledge on complex dystonias with a particular view of recent developments with respect to advances in diagnosis and management, including causative treatments.

## Introduction

Dystonia is thus currently defined as “a movement disorder characterized by sustained or intermittent muscle contractions causing abnormal, often repetitive movements or postures, or both.” According to the new classification, the term “complex dystonia” is used for dystonias accompanied by neurologic or systemic manifestations beyond movement disorders. It encompasses a broad spectrum of syndromes and diseases that typically gradually evolve over time in the clinical course (Klein and Munchau 2013).

There are some characteristic dystonia features indicative of complex dystonia including sustained dystonia at rest rather than action-associated or action-specific occurrence typical for isolated or combined dystonia, and prominent tongue or perioral involvement leading to what has been labelled “*risus sardonicus*” (Klein et al. 1993). Typical additional signs accompanying complex dystonias are developmental delay or cognitive impairment, spasticity, ataxia, bulbar involvement including anarthria, visual impairment, oculomotor disturbances, hearing loss, or seizures (Klein and Munchau 2013).

Rational diagnostic workup and early diagnosis in complex dystonias associated with marked disability are very important for two main reasons. First, some forms of complex dystonia are treatable, so that adequate and early therapy is mandatory.

Second, as is the case also for other patients with hitherto unexplained diseases, quality of life of caregivers, particularly mothers of affected children or adolescents, significantly improves by clarifying the etiologic, usually genetic, background of a disabling disease (Lingen et al. 2016). This is probably explained by reductions of the feeling of guilt in parents, who often implicitly or explicitly fear that they are responsible for the disease of their child (Lingen et al. 2016).

With this review, we aim to highlight the important role of interdisciplinary care for patients with complex dystonias that are prototypes of rare diseases. This field advances rapidly, particularly with respect to diagnosing and treating patients, so that there is a need for a systematic summary of the current state of knowledge.

## Complex dystonias in children

After tics, dystonia is the second most common movement disorder in children (Baumer et al. 2017). In contrast to dystonia in adult patients, which is typically isolated or combined and focal or segmental, dystonia in children is complex in about 50% of cases and tends to generalize (Marsden and Harrison 1974). In fact, in 80% of patients with generalized dystonia symptoms started before the age of 15 (Marsden and Harrison 1974). The earlier symptoms begin, the higher is the probability that they worsen over time (Burke et al. 1986a). Patients with onset in the legs tend to have a more rapid spread of dystonia to other body regions than those, in whom dystonia starts in the arms (Greene et al. 1995). As regards etiology, genetically determined forms and acquired forms, including perinatal injury, are much more frequent in children than in adults, wherein the majority of cases etiology is unknown, even though a genetic cause is often suspected.

### Acquired forms

#### Infantile cerebral palsy (CP)

Infantile cerebral palsy is the most frequent cause of acquired dystonias in children (Lin et al. 2014). The term comprises different syndromes, all resulting from brain damage for various reasons, including stroke and infection during pregnancy or perinatally. Many children also have other symptoms and signs in addition to dystonia, particularly spasticity, cognitive impairment, or epilepsy, thus qualifying to be grouped into the category of complex dystonias. In these cases, great caution is necessary not to overlook potentially treatable hereditary diseases (MacLennan et al. 2015), e.g., l-Dopa-responsive dystonia that can present as complex dystonia and be misinterpreted as CP (Giri et al. 2019; Pearson et al. 2019).

Although the classification of CP is a matter of ongoing debate, its core components are clear. Thus, it is a disorder of movement and posture caused by brain lesions that may be microscopic, e.g. hypoxia leading to selective necrosis of neurons often not captured by conventional brain imaging. It is acquired early in life and typically does not progress (Pakula et al. 2009). Onset though may be delayed, for instance in dyskinetic CP with symptoms becoming apparent in the first year of life, but not immediately after birth. Dystonic CP is dominated by abnormal postures that are often less dynamic and variable compared to isolated dystonias (Christine et al. 2007). In many cases, lingual, pharyngeal, and laryngeal muscles are affected leading to speech- and swallowing difficulties. MR-imaging typically shows leukoencephalopathy or lesions in globus pallidus or thalamus, but may also be unremarkable (Benini et al. 2013). Importantly, whenever there is a discrepancy between clinical phenomenology and brain imaging, e.g., a severe or complex syndrome with generalized dystonia, spasticity, and cognitive impairment associated with normal brain imaging or only subtle changes, causes other than acquired lesions have to be considered, particularly genetically determined autosomal recessive or de novo diseases (Zouvelou et al. 2019; Pearson et al. 2019).

#### Bilirubin encephalopathy

A special form of brain damage, that can clinically present as CP, is bilirubin encephalopathy (Rose and Vassar 2015). Particularly, the globus pallidus and nucleus subthalamicus have a predilection for being damaged by hyperbilirubinemia (Gkoltsiou et al. 2008). The etiology of hyperbilirubinemia is manifold, i.e., hemolytic disorders. Examples for specific etiologies are AB0-incompatibility, genetic diseases including beta-thalassemia and Glucose-6-phosphate dehydrogenase deficiency, birth asphyxia, and congenital infections, i.e., cytomegalovirus infection and syphilis (Dennery et al. 2001; Olusanya et al. 2015). The prevalence and disease burden of neonatal hyperbilirubinemia is higher in developing countries due to the lack of routine treatment, diagnostic delays, limited accessibility to medical care, and financial/cultural constraints (Olusanya et al. 2014).

In the acute phase, newborns are hypotonic, have feeding problems, and often epileptic seizures. Additionally, they can show a characteristic high-pitched screaming. This is followed by fever, generalized hypertonia including opisthotonus, upward gaze palsy, and auditory dysfunction (Shapiro 2005). The term “Kernicterus” designates the long-term outcome of acute bilirubin encephalopathy. It is typically evident after  one year of age. Clinical characteristics are hypertonia, movement disorders, auditory processing disturbance with or without hearing loss, oculomotor impairments, particularly restriction of upward vertical gaze, and dysplasia of the enamel of deciduous teeth (Shapiro 2010). First-line treatments in the acute phase are phototherapy and exchange transfusions to avoid irreversible damage (Shapiro and Riordan 2020).

#### Basal ganglia calcification

Asymptomatic calcifications are common in adults, especially in older people (Yamada et al. 2013). In children with complex dystonia, brain calcifications are an important clue to the underlying cause, but they are not specific (Legido et al. 1988). They can be associated with intracerebral infection (e.g., cysticercosis or toxoplasmosis), immunologic diseases (e.g., systemic lupus erythematosus), metabolic disorders (e.g., hypoparathyroidism), or mitochondrial diseases (Donzuso et al. 2019). There are also defined monogenic diseases causing basal ganglia calcification (see Table 1) (Tonduti et al. 2018).

**Table 1:** Genetic causes of complex dystonias

	Disease	Gene (MOI)	Characteristics / diagnostic clues
Typically childhood-onset	Rett (like)-syndrome	*MECP2, FOXG1, GNB1* (XL)	Autism, unusual stereotypies after a short episode of normal development
	Westphal variant of HD	*HTT* (AD)	Positive family history, often paternal transmission MRI: caudate atrophy
	Disorders of monoamine neurotransmitter metabolism	*GCH1* (AD/AR), *PTPS* (AR), *SPR* (AR), *AADC* (AR), *TH* (AR)	Developmental delay, epilepsy, dystonia with diurnal fluctuations; oculogyric crises; newborn screening/metabolite abnormalities in blood/urine/CSF
	GLUT1-deficiency	*SLC1A2* (AD)	Epilepsy, paroxysmal exertional dystonia
	CHOR/DYT-ADCY5	*ADCY5* (AD)	Paroxysmal nocturnal dyskinesia; perioral chorea and myoclonus
	Dystonia/parkinsonism with manganese accumulation (DYT/PARK-SLC30A10)	*SLC30A10* (AR), *SLC39A14* (AR)	Polycythemia and liver cirrhosis (SLC30A10); T1-weighted hyperintensities in basal ganglia and cerebellum
	Dystonia-deafness-optic neuronopathy syndrome	*TIMM8A* (XL)	Oromandibular dystonia and deafness
	Other dystonia-deafness-syndromes	*SERAC1* (AD), *SUCLA2* (AD), *DDP* (XL)	Dystonia, deafness, liver failure
	Organic acidurias	*L2HGDH* (AR), *GCDH* (AR)	Acute encephalopathic crises triggered by fever or vaccination
	Aminoacidurias	*CBS* (AR), *PAH* (AR),	Failure to thrive, metabolites in urine CBS: various eye conditions PKU: light eye/skin/hair
	Biotinidase deficiency	*BTD* (AR)	Seizures, hypotonia; reduced biotinidase activity; newborn screening
	Biotin-thiamine-responsive basal ganglia disease	*SLC19A3* (AR)	Recurrent subacute encephalopathy often triggered by fever; MRI: symmetric bilateral edematous lesions in basal ganglia and cortex
	Galactosemia	*GALT* (AR)	Hepatobiliary disease, vomiting, global developmental delay, cataract
	Aicardi–Goutieres syndrome	*ADAR1, RNASEH2A, RNASEH2B, RNASEH2C, SAMHD1, TREX1* (AR/AD)	Early-onset encephalopathy, chilblain lesions; MRI: basal ganglia calcification, leukoencephalopathy; Lymphocytosis in CSF
	KMT2B-associated dystonia	*KMT2B* (AD)	Progressive dystonia, onset in lower limb; commonly associated with developmental delay and microcephaly
	Leigh syndrome	Pathogenetic variants in mtDNA; nuclear genes (AR, XL) For details, see (Schubert Baldo and Vilarinho 2020)	Developmental regression, bulbar signs; MRI: symmetric necrotic lesion in the basal ganglia, cerebellum, thalamus, brain stem, and optic nerves
	MELAS	Pathogenetic variants in mtDNA (*MT-TL1, MT-TQ, MT-TH, MT-TK, MT-TC, MT-TS1, MT-TS2, MT-ND1, MT-ND5, MT-ND6*)	Stroke-like episodes, lactate acidosis, mitochondrial myopathy; MRI: white matter lesions
Typically adult-onset	Neuro-acanthocytosis	*VPS13A1* (AR, possibly AD)	Lingual dystonia; intermittent head drop; acanthocytes in blood smear, elevated CK
	Primary familial brain calcification	*PDGFB* (AD), *PDGFRB* (AD), *SLC20A2* (AD), *XPR1* (AD), *MYORG* (AR)	Calcifications in basal ganglia, white matter, and cerebellum
	Wilson’s disease	*ATP7B* (AR)	Hepatobiliary disease, Kaiser-Fleischer corneal ring, low ceruloplasmin plasma level
	NBIAs (see Table 2)	*FTL* (AD), *CP* (AR)	MRI-abnormalities predominantly in the basal ganglia
	Leber’s hereditary optic neuropathy “plus”	Pathogenetic variants in mtDNA (*MT-ND1, MT-ND2, MT-ND4, MT-ND4L, MT-ND5, MT-ND6, MT-CYB, MT-CO1, MT-CO3, MT-AP6*)	Optic nerve changes in fundoscopy
	*POLG*-related disorders	*POLG* (AD/AR)	Progressive external ophthalmoplegia, ataxia, neuropathy; hepatobiliary disease
Variable onset	DRPLA	*ATN1* (AD)	Juvenile: myoclonic epilepsy and intellectual deficits
			Adult: ataxia, chorea, myoclonus, dementia
			Symmetrical, diffuse white matter, thalamic and pallidal lesions on T2-weighted images; atrophy of cerebellum and pontine tegmentum
	NBIAs (see Table 2)	*CP* (AR), *FLT1* (AD), *PANK2* (AR), *COASY* (AR), *PLA2G6/iPLA2* (AR), *C19orf12* (AR), *FA2H* (AR), *WDR45* (XL), *CRAT* (AR), *REPS1* (AR), *AP4M1* (AR), *GTPBP2* (AR)	Iron accumulation predominantly in the basal ganglia
	Niemann-Pick type C	*NPC1, NPC2* (AR)	Supranuclear gaze palsy, splenomegaly, increased oxysterol blood levels
	GM1/GM2-Gangliosidosis	*GLB1, GM2A* (AR)	Infantile/juvenile: severe mental retardation, seizures, spastic tetraparesis
			Adult: dysarthria, gait disturbance
			MRI: hyperintensity of caudate nucleus and putamen

*AD* autosomal dominant, *AR* autosomal recessive, *CBS* Cystathionin-beta-synthetase, *CSF* cerebrospinal fluid, *DRPLA* dentato-rubro-pallido-lysian atrophy, *GCDH* Glutaryl-CoA dehydrogenase, *GLUT1* Glucose transporter 1, *HD* Huntington’s disease, *mtDNA* mitochondrial DNA, *MELAS* mitochondrial encephalopathy with lactic acidosis, and stroke-like episodes, *MOI* mode of inheritance, n.a.: not available, *NBIA* neurodegeneration with brain iron accumulation, *PKU* phenylketonuria, *XL* X-linked

### Monogenic diseases

Many genetically determined diseases presenting with complex dystonias commence in childhood or adolescence. An overview is given in Table 1. Diseases that are clinically particularly relevant are described in the following section.

#### Disorders of monoamine neurotransmitter metabolism

Pathogenic variants in the *sepiapterin reductase (SPR)*, *tyrosine hydroxylase* (*TH*), and *6-pyruvoyl tetrahydrobiopterin synthase* (*PTPS*) gene cause deficient synthesis of dopamine (and other monoamine acids), resulting in complex dystonia (Table 1). In contrast to heterozygous pathogenic variants in the *guanosine triphosphate cyclohydrolase 1* (*GCH1*) gene, which cause DYT/PARK-GCH1 (Segawa syndrome), the prototype disorder of Dopa-responsive dystonia, typically presenting as combined dystonia, homozygous variants result in complex dystonia syndromes. Different from autosomal-dominant DYT/PARK-*GCH1* dystonia, the other forms are only partially responsive to L-Dopa and require additional treatment such as tetrahydrobiopterin, 5-hydroxytryptophan, or other dopaminergic medication (Opladen et al. 2020). Patients often have oculogyric crises, hypotonia, global developmental delay, and sometimes epilepsy in addition to dystonia. These diseases usually start between birth and 6 years, i.e., earlier than DYT/PARK-*GCH1*. Some are associated with hyperphenylalaninemia, which is detected in newborn screening in many countries. Many forms show distinct patterns of abnormalities of pterins and other neurotransmitters in blood, cerebrospinal fluid (CSF), and/or urine.

#### Paroxysmal dyskinesias

Whereas patients with paroxysmal kinesigenic or non-kinesigenic dyskinesia usually present with combined dystonia, paroxysmal exertional dystonias (PED) can occur as complex dystonia. PED is characterized by attacks of dystonia triggered by prolonged physical activities. The most recognized monogenic form of PED is the Glut1-deficiency syndrome, caused by mutations in the *SLC1A2* gene, which may present with variable combinations of spasticity, ataxia, seizures, and intellectual disability in addition to paroxysmal dystonia (Pons et al. 2010). A timely start with a ketogenic diet or modified Atkin’s diet can lead to dramatic improvement and is associated with an improved long-term clinical outcome (Alter et al. 2015; Amalou et al. 2016; Sandu et al. 2019).

Another recently described paroxysmal movement disorder, sometimes manifesting with dystonia, is CHOR/DYT-*ADCY5* (Chen et al. 2012). Severe phenotypes are clinically complex and can present with developmental delay, hypotonia, chorea, and myoclonus (not myokymia) (Tunc et al. 2017). A characteristic feature is exacerbation or attacks of dyskinesia during drowsiness or during sleep without abnormalities in the electroencephalography (Chang et al. 2016).

#### Neurodegenerative diseases

Complex dystonias that after a period of normal development evolve gradually with progressive symptoms suggest an underlying disease leading to neurodegeneration. Clinical red flags pointing in this direction are prominent bulbar involvement, rapid progression of dystonia, oculomotor signs, associated hearing problems or vision loss, or cognitive decline and behavioral abnormalities (Schneider and Bhatia 2010). The list of possible causes is very long. Children presenting with complex dystonia often need comprehensive diagnostic workup including brain imaging, CSF examination, ophthalmological assessment, and investigation of organic acids.

Typical examples are diseases belonging to the group of neurodegeneration with brain iron accumulation (NBIAs, see below) and Westphal’s variant of Huntington’s disease. The latter is particularly relevant, because, in contrast to adult patients, it typically presents with a combination of dystonia, parkinsonism, cognitive dysfunction but not necessarily chorea (Letort and Gonzalez-Alegre 2013) and is therefore often overlooked or diagnosed late. Of note, even if there is no strict correlation (Quarrell et al. 2013), Huntington’s disease manifests in childhood or adolescence when CAG-repeat numbers are high, which is typically the case when the disease is inherited paternally (Went et al. 1984). Potentially treatable diseases such as Niemann-Pick type C (see below) and ataxia with vitamin E deficiency should be considered, because early diagnosis and treatment are associated with better long-term outcomes.

#### Biotin- and thiamine-responsive basal ganglia disease

Biotin- and thiamine-responsive basal ganglia disease is an autosomal recessive inherited disease caused by biallelic mutations in the *SLC19A3* gene, which encodes for the thiamine transporter-2. The disease manifests with acute or subacute encephalopathy. It is characterized by dystonia, confusion, dysarthria, external ophthalmoplegia, ataxia, and seizures. Typical triggers are febrile illnesses (Alfadhel et al. 2013). Brain imaging frequently is a key to diagnosis, showing central bilateral necrosis in the head of the caudate nucleus and the putamen (Tabarki et al. 2013). If untreated, the prognosis is grave with the development of severe generalized dystonia and parkinsonism, quadriplegia, and epilepsy. The mortality is high (Tabarki et al. 2013). An early diagnose is very important, as sufficient treatment with thiamine and biotin can lead to significant clinical improvement (Tabarki et al. 2015).

## Complex dystonias in adults

### Acquired forms

There are three main scenarios of acquired dystonia in adults.

First, focal brain lesions, predominantly affecting the putamen, the thalamus, or globus pallidus, e.g. following stroke, inflammation, or surgical interventions. They can lead to contralateral hemidystonia (Munchau et al. 2000) and typically develop month or even years after the brain damage has occurred. The reason for this delay in onset is unclear, although aberrant reorganization has been hypothesized (Hinkley et al. 2009). Whereas hemidystonia can dominate the clinical picture, it is rarely the only sign. Still, it is in fact often accompanied by spasticity, sensory disturbances, particularly diminished joint position sense, or cognitive impairment, and can, therefore, be classified as complex dystonia in most cases.

Second, drugs, particularly antipsychotic medication, can cause tardive dystonia. It mostly manifests as craniocervical or oromandibular dystonia that is typically isolated or combined, but can also be complex considering that many afflicted patients also have cognitive impairment (Pourcher et al. 1993).

Third, functional movement disorders often present as dystonia and are typically accompanied by other symptoms and clinical signs (Thenganatt and Jankovic 2015), thus also qualifying to be categorized as complex dystonia.

### Dystonia in Parkinson and atypical Parkinson’s disease

Parkinson syndromes can present with dystonia. Patients with classical Parkinson’s disease can manifest with dystonia, typically affecting the feet or legs (Shetty et al. 2019), and may also develop sometimes painful dystonia in the extremities. Obviously, dystonia in patients with parkinsonism is usually not the leading sign, but can give diagnostic clues and can be troublesome. Apart from its occurrence in classical Parkinson’s disease, where it would most appropriately be categorized as combined, dystonia in other, i.e., atypical Parkinson syndromes, can be classified as complex. Thus, in patients with multiple system atrophy, facial or cervical dystonia is quite common (Thongchuam et al. 2020). Patients with progressive supranuclear palsy can have brachial dystonia or apraxia of eyelid opening (Armstrong 2011; Barclay and Lang 1997), which is considered a form of dystonia by some researchers (Krack and Marion 1994). Dystonic posturing along with rigidity and cortical sensory deficits are cardinal features of cortico-basal syndromes (Armstrong et al. 2013).

### Monogenic diseases

Most monogenic complex dystonias occurring in adulthood are neurodegenerative diseases. They typically involve the basal ganglia and are sometimes associated with brain calcification or accumulation of metals, particularly copper, iron, and manganese. Brain imaging is often very helpful to identify a disease or disease-group. An overview is given in Table 1.

#### Wilson’s disease

Wilson’s disease is a representative example of a treatable genetically determined disease where metal deposition plays a crucial role. In Wilson’s disease, copper accumulates in the liver and central nervous tissue due to a biallelic mutation in the *ATP7B* gene. Clinical features are variable. Typically, the disease starts in adolescence or early adulthood as a hepatopathy, followed by neurological complications (Mulligan and Bronstein 2020). Diagnostic delay is longer in patients presenting with neuropsychiatric symptoms (Merle et al. 2007). Rarely, the disease commences later in life. Cases with onset in the 7th and 8th decades have been described (Ala et al. 2005). The most common neurologic features are bulbar signs, including dysarthria and dysphagia, gait abnormalities, parkinsonism, tremor, and dystonia, typically prominently involving the face leading to *risus sardonicus* (Machado et al. 2006; Burke et al. 2011). 30–40% of patients have neuropsychiatric abnormalities, including apathy, indifference, and sometimes psychosis (Zimbrean and Schilsky 2014). Patients with neurological or neuropsychiatric signs have a pathognomonic Kayser–Fleischer corneal rings, i.e., copper deposits in the corneal membrane. In most patients, structural abnormalities can be identified on MRI, predominantly T2-hyperintensities involving the striatum and globus pallidus (Hermann 2014). The diagnosis can be confirmed by laboratory testing of 24 h urine copper assay. Investigations of free serum copper and serum coeruloplasmin can be used as screening tests. In predominantly hepatic forms, liver biopsy is the gold standard. Genetic confirmation is recommended and early treatment is essential. The mainstay of treatment is the chelation of copper, which should be started gradually given the risk of worsening symptoms initially presumably due to copper mobilization, particularly in patients with neurologic symptoms (Weiss et al. 2013). A novel oral copper-protein-binding agent, bis-choline tetrathiomolybdate is currently under investigation (Weiss et al. 2018).

## Complex dystonias with variable onset

### Genetically determined diseases

#### Niemann–Pick type C

Niemann–Pick type C is caused by an autosomal recessive inherited mutation in the *NPC1* or *NPC2* gene, leading to impaired function of proteins involved in the lipid transport from cells to the extracellular matrix. Thus, toxic cholesterol accumulation causes cellular damage (Xu et al. 2019). Onset age varies significantly from the newborn period up to late adulthood (Bajwa and Azhar 2020). Typically, the disease begins in middle or late childhood. The classic phenotype is a gradually developing neuropsychiatric syndrome. It consists of frontal signs, in combination with predominantly upper body, generalized dystonia, a pan-cerebellar syndrome, and supranuclear vertical gaze palsy. These symptoms are often accompanied by an asymptomatic hepato-splenomegaly (Wijburg et al. 2012; Sedel 2010). Early diagnosis is important, because therapy with Miglustat can halt progression (Patterson et al. 2020).

#### Neurodegeneration with brain iron accumulation (NBIA)

NBIAs are an expanding group of progressive diseases characterized by abnormal accumulation of iron in the brain, particularly the basal ganglia, caused by different mutations with different modes of inheritance (see Table 2; Akcakaya et al. 2019; Mari et al. 2018; Rattay et al. 2019; Haack et al. 2012; Marchi et al. 2019) leading to neurodegeneration. Most patients present with movement disorders, particularly dystonia and parkinsonism. Dysarthria, spasticity, neuropsychiatric abnormalities, polyneuropathy, or visual loss are common in some forms. Onset age and progression are very variable (Gregory and Hayflick 1993). Although causative genes are involved in different biochemical pathways, brain iron accumulation is a defining hallmark. Relevant pathophysiologic mechanisms include iron oxidation deficit, free, i.e., non-transferrin bound, toxic iron, oxidative stress, lysosomal or mitochondrial dysfunction, irregular calcium homeostasis, membrane remodeling, and autophagy (Levi et al. 2019; Santambrogio et al. 2015,2020; Brissot et al. 2012; Maccarinelli et al. 2015; Seibler et al. 2018; Stelten et al. 2019). Magnetic resonance imaging, particularly iron sensitive sequences, is very helpful, because most NBIAs have characteristic patterns of abnormalities (Lehericy et al. 2020).

**Table 2:** Overview of Neurodegenerations with Brain Iron Accumulation (NBIA)

Disease	Gene (Inheritance)	Function	Onset	MRI-findings	Diagnostic clues	Causal treatment
Aceruloplasminemia NBIA/DYT/PARK-CP (Marchi et al. 2019)	*CP* (AR)	Cu-dependent ferroxidase	Adulthood	Iron accumulation in striatum, dentate nucleus, and thalamus	DM, microcytic anemia, undetectable serum ceruloplasmin levels	Iron-chelating therapy
Neuroferritinopathy NBIA/CHOREA-FTL (Kubota et al. 2009)	*FTL1* (AD)	Iron storage	Adulthood	Lesions in globus pallidus, putamen, and dentate nucleus	Decreased level of serum ferritin	n.a
PKAN NBIA/DYT-PKAN2 (Kurian and Hayflick 2013)	*PANK2* (AR)	Coenzyme A synthesis; fatty acid metabolism	Infancy	“Eye-of-the tiger”-sign	MRI sign	n.a
CoPAN (Evers et al. 2017)	*COASY* (AR)	Coenzyme A synthase	Infancy	Iron deposit in globus pallidus and substantia nigra	Similar to PKAN	n.a
INAD NBIA/DYT/PARK-PLA2G6 (Gregory et al. 1993)	*PLA2G6, iPLA2* (AR)	Arachidonic acid release	Typical: infancy	Iron deposit in globus pallidus, cerebellar atrophy	Typical: neuropathy with spheroidal bodies	n.a
			Atypical: adulthood		Atypical: dystonia-parkinsonism with psychosis and dementia	
MPAN HSP/NBIA-C19orf12 (Akcakaya et al. 2019)	*C19orf12* (AR)	Unknown	Childhood	Iron deposit in globus pallidus and substantia nigra	Dystonia, neuropathy, optic atrophy	n.a
FAHN HSP/NBIA-FA2H (Mari et al. 2018; Rattay et al. 2019)	*FA2H* (AR)	Fatty acid hydroxylase	Childhood	Iron deposit in globus pallidus, white matter changes, cerebellar atrophy, thinning of corpus callosum	Spasticity, ataxia, dystonia, optic atrophy, bristle-like hair	n.a
BPAN NBIA/PARK-WDR45(Haack et al. 2012)	*WDR45* (XL)	Autophagy	Childhood	Iron deposition in the globus pallidus and substantia nigra	Rett-like syndrome, stereotypies	n.a
Rare forms	*CRAT, REPS1, AP4M1, GTPBP2* (all AR)					

*AD* Autosomal dominant, *AR* Autosomal recessive, *BPAN* Beta-propeller-protein-associated neurodegeneration, *CoPAN* COASY protein-associated neurodegeneration, *DM* Diabetes mellitus, *FAHN* Fatty Acid Hydroxylase-Associated Neurodegeneration, *INAD* Infantile Neuroaxonal Dystrophy, *MPAN* Mitochondrial Membrane Protein-Associated Neurodegeneration, *n.a.* Not available, *PKAN* Pantothenate kinase-associated neurodegeneration, *XL* X-linked

Pantothenate kinase-associated neurodegeneration (PKAN; NBIA/DYT-PKAN2) is the prototype and most frequent NBIA. 35–50% of NBIAs are caused by mutations in the *PANK2* gene (Hayflick et al. 2003). Typically, onset is in early childhood. Symptoms gradually progress and contain spastic-dystonic paraparesis with a tendency for dystonia to generalize, followed by dysarthria, dysphagia, and sometimes parkinsonism and behavioral problems (Kurian and Hayflick 2013). Cognition though is often intact. There is a pathognomonic “eye-of-the-tiger-sign” on T2-weighted images caused by focal iron accumulation in the globus pallidus, which occasionally disappears in the course of the disease (Baumeister et al. 2005). The pathophysiology remains unclear, but mitochondrial dysfunction and impaired calcium homeostasis are discussed to play a pivotal role (Santambrogio et al. 2015, 2020). Treatment with deferiprone is still under investigation, but appears to slow disease progression with a good safety profile (Klopstock et al. 2019; Rohani et al. 2017; Zorzi et al. 2011). Deep brain stimulation (DBS) of the globus pallidus internus can significantly improve dystonia (Svetel et al. 2019; Timmermann et al. 2010).

## Diagnostic approach to complex dystonias

The way to a specific diagnosis for patients with complex dystonias is often long. A clear diagnostic pathway can help to prevent diagnostic delays and thus reduce disease burden for affected patients. Given that complex dystonias can occur in diseases affecting different organs in addition to the nervous system, for instance, spleen and liver in Niemann-Pick type C, can manifest in childhood, adolescence and adulthood and are often genetically determined, expertise from different specialties may be required. This calls for specialized centers bringing together different disciplines, which is realized, for instance, in centers for rare diseases in the context of European Reference Networks (ERNs) for rare diseases and is also facilitated by the German Academy for Rare Neurological diseases (Munchau et al. 2019). Core facilities should comprise regular multi-disciplinary case conferences according to standardized protocols. Ideally, web-based case conferences fulfilling data protection rules should also be offered. One example is the European Clinical Patient Management System (CPMS) allowing to include experts from national or international partner centers to augment diagnostic and management expertise (Smith et al. 2020). It also offers the opportunity to use virtual communication tools and DICOM viewers. The system is not only used for neurological purposes, but also in many other disciplines. If patients are sent to centers with an experienced multi-disciplinary team, this conveys benefits regarding classification, diagnostic yield, and targeted treatment strategies (van Egmond et al. 2018).

**Fig. 1:**
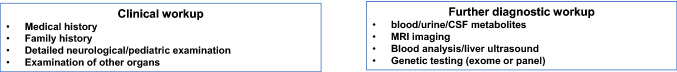
Proposal for diagnostic workup in suspected complex dystonias

The crucial starting point of any diagnostic route is thorough clinical assessment, including family history, concomitant diseases, and organ involvement. Based on clinical acumen, further diagnostic workup is tailored. A diagnostic workup is proposed in Fig. 1.

**Fig. 2:**
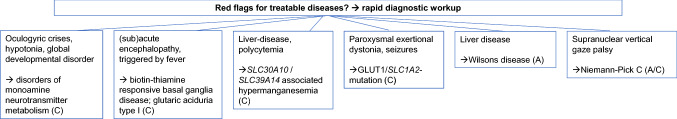
Red flags for treatable diseases with complex dystonia; A = onset in adulthood; C = childhood-onset

“Red flags” for treatable diseases should always be kept in mind (see Fig. 2). In children, metabolic testing should be initiated parallel to neuroimaging. If a monogenic cause is suspected, diagnostic gene panels are usually preferred over single gene sequencing unless there is a high degree of suspicion for a certain disease (van Egmond et al. 2017). Whole-exome and whole-genome sequencing should be considered, particularly in cases with unusual phenotypes or suspected de novo mutations. A pre-requisite for these investigations is close cooperation between treating physicians and (neuro-)geneticists, including regular case conferences.

## Therapeutic and long-term management

If causative treatment is available, it should be initiated as soon as possible to improve long-term outcome and prevent unnecessary complications. Especially in inborn errors of metabolism, an early diagnosis and treatment is highly important for a good outcome (Ebrahimi-Fakhari et al. 2019). Disease-specific treatments are outlined in Table 3. In some diseases, treatments have become standard even in the absence of randomized clinical trials, as is the case, for example, for l-Dopa therapy in Dopa-responsive dystonia.

**Table 3:** Overview of treatable forms of complex dystonia

Disease	Causal treatment
Disorders of monoamine neurotransmitter metabolism	Supplementation with tetrahydrobiopterin, L-Dopa, 5-hydroxytryptophan, or other dopaminergic medications
GLUT1-deficiency	Ketogenic diet Triheptanoin
Dystonia/parkinsonism with manganese accumulation (DYT/PARK-SLC30A10)	EDTA-chelation therapy Iron-therapy
Glutaryl-CoA-dehydrogenase-deficiency (GCDH)	Dietary lysin-restriction L-Carnitine supplement Prevention and management of known triggers
Cystathionine beta-synthase deficiency (CBS)	Vitamin B1/B6/B12 supplement Dietary restriction of methionine/betaine
Phenylketonuria	Dietary restriction of phenylalanine
Biotinidase deficiency	Oral biotin supplements
Galactosemia	Specific diet free of galactose and lactose
Wilson’s disease	Copper-chelating agents
Leber’s hereditary optic neuropathy “plus”	Idebenone
Niemann-Pick type C	Miglustat

*GLUT1* glucose transporter 1

Given the rarity of many diseases associated with complex dystonias, evidence for the efficacy of a given medication is often very limited, so that rational treatment strategies for these diseases often have to be based on small clinical trials, expert opinion, or consensus statements (Jinnah et al. 2018).

In many patients with complex dystonia, symptomatic therapy is still the only option. Even if causative treatment is available, many patients still need additional support. Long-term personalized treatment should be planned and implemented in specialized multi-disciplinary centers, including physiotherapists, speech therapists, psychologists, and health care professionals in addition to neurologists and pediatric neurologists to support affected patients and their families, caregivers, and local physicians. Whenever possible patients should be included in disease registries and ongoing clinical trials necessitating close cooperation of specialized centers in national and international networks such as the ERN (see above) (Smith et al. 2020). Special transition services for young adults from neuro-pediatric to adult neurologic specialty centers should be offered or developed.

### Oral medication

Oral medication is recommended for segmental and generalized dystonia. Particularly, trihexyphenidyl and baclofen can have a positive effect (Woo et al. 2020). The response to oral medication is variable.

#### Trihexyphenidyl

Trihexyphenidyl is an anticholinergic drug, which caused a significant benefit at a mean follow-up of 2.4 years in an early study (Burke et al. 1986b). The dose can slowly be increased, while side effects should be monitored, particularly sedation, dry mouth, blurred vision, and cognitive impairment (Taylor et al. 1991).

#### Baclofen

Baclofen is a pre-synaptic gamma-aminobutyric acid agonist. It can be used in combination with trihexyphenidyl for additional benefit. It has to be kept in mind that the main side effect is a general decrease in muscle tone, which can cause or increase pre-existing paresis and hypotonia. Additionally, sedation and dizziness can occur as side effects. If the medication is stopped, it must be tapered slowly to avoid withdrawal seizures (Thenganatt and Jankovic 2014).

#### Other oral medications

Tetrabenazine can improve dystonia, but is most frequently used for tardive dyskinesia.

l-Dopa is the mainstay of treatment in l-Dopa-responsive dystonias, but can also be tried in other forms of childhood-onset dystonia (Kitahara et al. 2009). Other agents shown to be effective in small case series or small trials include clonazepam, anticonvulsants, or muscle relaxants and can be tried to treat refractory dystonias (Thenganatt and Jankovic 2014).

### Botulinum neurotoxin

Botulinum neurotoxin causing local muscle weakness due to peripheral chemical denervation is recommended as a first-line treatment for isolated focal and segmental dystonias (Albanese et al. 2011). Still, it can also be very effective in other forms of dystonia. Most patients have a long-term response after repeated treatment cycles (Ramirez-Castaneda and Jankovic 2014).

### Intrathecal baclofen

Intrathecal baclofen often leads to significant improvement in children with severe spasticity and improves dystonia (Stewart et al. 2020). It is an established option in patients with treatment-refractory lower limb dystonia and spastic paraparesis. The main problems of this treatment are weakness, pump failure, or surgical complications. Treatment with intrathecal baclofen requires a specialized team to manage possible complications (Brennan and Whittle 2008).

### Deep brain stimulation (DBS)

DBS of the internal globus pallidus is commonly used as a surgical treatment for various forms of generalized, segment, or focal dystonia (Tsuboi et al. 2020). Given the rarity of most complex dystonias, evidence for its usefulness in these patients is limited. DBS should be considered in therapy refractory cases, but should only be carried out in experienced centers after a careful risk–benefit assessment. Additionally, cases should be included in prospective clinical registries (Artusi et al. 2020).

## Conclusions

In this review, we have highlighted the crucial role of interdisciplinary and cross-professional cooperation in the care of patients with complex dystonias with a special focus on the identification of treatable diseases. Prominent examples are disorders of monoamine neurotransmitter metabolism, Glut1-deficiency syndrome, Niemann–Pick Type C, and Wilson`s disease. Future perspectives include an increasing role for the translation of insights from basic science regarding defined, often novel diseases, to clinical care, advances in early diagnosis by means of widely available online tools, and progress in digital case conferences. The development of specialized centers, mutually beneficial and dynamic networks for rare diseases, clinical registries, and structured training for rare diseases are pre-requisites for rational and personalized management of patients with complex dystonias.

## Abbreviations

DBS: Deep brain stimulation
CP: Infantile cerebral palsy
CPMS: Clinical Patient Management System
CSF: Cerebrospinal fluid
ERN: European Reference Networks
NBIA: Neurodegeneration with brain iron accumulation
PED: Paroxysmal exertional dystonias
PKAN: Pantothenate kinase-associated neurodegeneration
